# Piperine potentiates the hypocholesterolemic effect of curcumin in rats fed on a high fat diet

**DOI:** 10.3892/etm.2014.1717

**Published:** 2014-05-16

**Authors:** YAOSHENG TU, DONGMEI SUN, XIAOHUI ZENG, NAN YAO, XUEJUN HUANG, DANE HUANG, YUXING CHEN

**Affiliations:** Traditional Chinese Medicine Pharmacological Research Office, Guangdong Provincial Institute of Traditional Chinese Medicine, Guangzhou, Guangdong 510095, P.R. China

**Keywords:** curcumin, piperine, cholesterol, rat, mRNA

## Abstract

It has previously been demonstrated that curcumin possesses a hypocholesterolemic effect and potentiates numerous pharmacological effects of curcumin, however, the mechanisms underlying this hypocholesterolemic effect and the interaction between curcumin and piperine remain to be elucidated. In the present study, male Sprague-Dawley rats were fed on a high-fat diet (HFD) to establish a hyperlipidemia (HLP) model. Co-administration of curcumin plus piperine was found to decrease the levels of total cholesterol (TC), triglyceride (TG) and low-density lipoprotein cholesterol in the serum and liver, as well as increase the levels of fecal TC, TG and total bile acid, compared with administration of curcumin alone. Curcumin plus piperine also markedly increased the levels of high-density lipoprotein cholesterol. Furthermore, compared with administration of curcumin alone, administration of curcumin plus piperine resulted in a significant upregulation of the activity and gene expression of apolipoprotein AI (ApoAI), lecithin cholesterol acyltransferase (LCAT), cholesterol 7α-hydroxylase (CYP7A1) and low-density lipoprotein receptor (LDLR). In conclusion, these results indicated that co-administration of curcumin plus piperine potentiates the hypocholesterolemic effects of curcumin by increasing the activity and gene expression of ApoAI, CYP7A1, LCAT and LDLR, providing a promising combination for the treatment of HLP.

## Introduction

Cardiovascular disease (CVD) is one of the major causes of mortality worldwide. An increase in blood total cholesterol (TC) levels, which is regulated by cholesterol absorption, synthesis, storage and excretion, is one of the major risk factors leading to the development of CVD (1). At present, the levels of TC in the serum under hyperlipidemic conditions may be lowered by diet or medication.

Curcumin, a hydrophobic polyphenol derived from the rhizome of the herb *Curcuma longa,* has been widely used as a spice and a coloring agent, and is a major ingredient of curry powder (2). However, several studies have demonstrated that curcumin also possesses anti-oxidant, anti-tumor and anti-inflammatory properties (3–11). In addition, previous studies on animals and humans have demonstrated that curcumin is capable of decreasing the levels of TC in the blood (12–16). However, curcumin has not yet been approved as a therapeutic agent for hyperlipidemia (HLP), mainly due to problems with its bioavailability (17).

Piperine is responsible for the pungency of spices gained from *Piper nigrum* and *Piper longum* (sources of black or long pepper) (18). Piperine is capable of affecting the metabolism of other substances, and if consumed with curcumin, it significantly increases the bioavailability of curcumin by inhibiting hepatic and intestinal glucuronidation (17,19,20). For example, piperine as an adjuvant increases the efficacy of curcumin by mitigating benzo(a)pyrene toxicity and enhancing the protective effect of curcumin against chronic unpredictable stress-induced cognitive impairment and depressive disorders (21–23).

A previous study investigated the effect of curcumin, piperine and quercetin in high-fat diet (HFD) and low-dose streptozotocin-induced diabetic rats; however, the study focused on glucose intolerance and oxidative stress (24). Therefore, whether piperine is able to affect the hypocholesterolemic effects of curcumin remains to be elucidated. The aim of the present study was to investigate the hypocholesterolemic effect of curcumin plus piperine on cholesterol metabolism in rats fed on a HFD.

## Materials and methods

### Materials

Curcumin and piperine were purchased from Sigma-Aldrich (St. Louis, MO, USA). TC, triglyceride (TG), high-density lipoprotein cholesterol (HDL-C), low-density lipoprotein cholesterol (LDL-C) and total bile acid (TBA) kits were purchased from Nanjing Jiancheng Bioengineering Institute (Nanjing, Jiangsu, China). The free cholesterol (FC) kit was purchased from Applygen Technologies Inc. (Beijing, China). Apolipoprotein AI (ApoAI) and ApoB kits were purchased from Shanghai Rongsheng Biotech Co., Ltd. (Shanghai, China). The bicinchoninic acid assay kit was obtained from Tiangen Biotech Co., Ltd. (Beijing, China). Ethanol and isopropanol of analytical grade were purchased from Dikma Technologies, Inc. (Beijing, China). TRIzol^®^ reagent was purchased from Invitrogen Life Technologies (Carlsbad, CA, USA). The RevertAid™ First Strand cDNA Synthesis kit and the Maxima™ SYBR-Green/Fluorescein qPCR Master mix (2X) were purchased from Thermo Fisher Scientific (Burlington, ON, Canada).

### Animals, diets and treatments

Thirty male Sprague-Dawley (SD) rats weighing between 180 and 220 g were purchased from the Guangdong Medical Laboratory Animal Center (Guangzhou, Guangdong, China). All rats were kept in a specific pathogen-free room under controlled conditions. A 12 h light-dark cycle was maintained, with lights on between 6:00 a.m. and 6:00 p.m., and the temperature was maintained at 23±1°C. The rats were provided with the standard laboratory diet and tap water and allowed to acclimatize to the environment for 1 week prior to the start of the experiment. Following 1 week, the rats were randomly divided into five groups with six rats in each group. The rats in the normal control (N) group were fed on a normal diet, whilst rats in the other groups were fed on a HFD, which contained 10% fat and 2% cholesterol for 8 weeks, in order to induce hypercholesterolemia. Following 5 weeks, with the exception of rats in the N group and the HFD control (H) group, rats were administered curcumin (100 mg/kg/day; C group), piperine (5 mg/kg/day; P group) or curcumin (100 mg/kg/day) plus piperine (5 mg/kg/day; CP group), dissolved in corn oil for 4 weeks. Rats in the N and H groups were orally administered corn oil for 4 weeks. At the end of 8 weeks all the rats were sacrificed in a 100% ether atmosphere and the blood, liver and feces were collected.

All experimental protocols were approved by the Animal Ethics Committee of Guangdong Provincial Institute of Traditional Chinese Medicine (Guangzhou, Guangdong, China). The care and treatment of the animals were conducted in accordance with the guidelines (publication no. 85-23, revised 1996) provided by the National Institutes of Health (Betheseda, MA, USA) and the U.S. Department of Agriculture (Washington, DC, USA).

### Serum sampling

Blood was collected from the abdominal aorta and left at room temperature for coagulation. The serum samples were obtained by centrifugation at 3,300 × g, 4°C for 10 min and stored at 4°C for further analysis.

### Liver sampling

Liver samples (0.3 g) from each rat were immediately removed and homogenized in phosphate-buffered saline (PBS; pH 7.2; 0.15 g/ml) at 4°C. The supernatant was centrifuged at 3,300 × g at 4°C for 10 min and stored at −80°C for analysis of the hepatic lipid levels. In addition, 0.1 g liver samples from each rat were immediately removed, washed with PBS, dried on filter paper and stored at −80°C for further analysis using polymerase chain reaction (PCR).

### Feces sampling

Feces from each rat were collected following 8 weeks for 3 days and dried at 60°C. Feces were then weighed and ground into a powder. A total of 0.5 g of feces powder from each rat was extracted three times with 10 ml 95% ethanol at 60°C and then filtered as well as extracted three times with 10 ml 95% ethanol at 60°C and then filtered. The residue was dissolved in PBS by sonication. The final solution was adjusted to a suitable concentration and frozen at −80°C for further analysis.

### Measurement of lipid levels in the serum, liver and feces

The serum, liver and fecal levels of TC, TG, HDL-C, LDL-C, ApoAI and ApoB were determined using the respective assay kits in accordance with the manufacturer’s instructions. All samples were detected using the 722 Grating Spectrometer (Shanghai Cany Precision Instrument, Co., Ltd., Shanghai, China).

### Measurement of TBA levels in feces

The TBA level in the feces extract was analyzed as previously described by Feldmann *et al* (25) using an assay kit in accordance with the manufacturer’s instructions. All samples were detected using the 722 Grating Spectrometer (Shanghai Cany Precision Instrument, Co., Ltd.).

### Measurement of lecithin cholesterol acyltransferase (LCAT) activity in serum

For the serum LCAT activity, the auto-matrix method was used to transfer 15 μl of serum into tubes A and B. Tube A was placed in an ice bath and tube B was placed at 37°C. The FC level in the two tubes was measured 60 min after using an assay kit in accordance with the manufacturer’s instructions. The LCAT activity was then calculated according to the decrement in non-esterified cholesterol by comparing the FC content in each tube. The LCAT activity was then determined using the previously described formula (26). All samples were detected using the 722 Grating Spectrometer (Shanghai Cany Precision Instrument, Co., Ltd.).

### Measurement of cholesterol 7α-hydroxylase (CYP7A1) activity in the liver

The rat liver microsomes were prepared as previously described (27–29). CYP7A1 activity in the rat liver was determined using high-performance liquid chromatography quantification of cholesterol metabolites, as described by Hylemon *et al* (30).

### Quantitative PCR (qPCR) analysis of hepatic mRNA levels

Total RNA was extracted from the rat liver samples using TRIzol reagent in accordance with the manufacturer’s instructions. A total of 3 μg of total RNA was reverse transcribed into cDNA using the First Strand cDNA Synthesis kit at 42°C for 1 h. qPCR was performed using the iQ5™ real-time PCR detection system (Bio-Rad, Hercules, CA, USA) and performed in a reaction mix containing 1 μl cDNA, 12.5 μl Maxima™ SYBR-Green/Fluorescein qPCR Master mix (2X), 1 μM forward primer and 1 μM reverse primer in a total volume of 25 μl. The cDNA was amplified using specific primers under the following conditions for 45 cycles: 94°C for 30 sec, an annealing temperature of 55°C for 30 sec and then 72°C for 50 sec, with a final incubation at 72°C for 7 min. The PCR primers used were as follows: ATP-binding cassette transporter A1 (ABCA1; GenBank accession no. NM_178095; 145 bp) forward, 5′-CAGCAACTACAGTGGCGGTAACA-3′ and reverse 5′-AATGCTTAGGGCACAATTCCACA-3′; ApoAI (GenBank accession no. NM_012738; 149 bp) forward, 5′-AAGGCATCTAAAGGTTGT-3′ and reverse 5′-TCAGGGTAGGGTGGTT-3′; CYP7A1 (GenBank accession no. NM_012942; 291 bp) forward, 5′-TGCCGTGTTGGTGAG-3′ and reverse 5′-TTCGCAGAAGTAGTGTAAT-3′; HMG-CoA reductase (HMGCR; GenBank accession no. NM_013134; 249 bp) forward, 5′-TGACGCTCTGGTGGA-3′ and reverse 5′-GTTACTGGGTTTGGTTTAT-3′; LCAT (GenBank accession no. NM_017024; 163 bp) forward, 5′-GCTACCGAAAGACAGAGG-3′ and reverse 5′-CCAAAGCCAGGGACA-3′; low-density lipoprotein receptor (LDLR; GenBank accession no. NM_175762; 117 bp) forward, 5′-CAGACCCAGAGCCATCGTAGTG-3′ and reverse 5′-GTCACCAGAGAGTAGATGTCTAC-3′; scavenger receptor B1 (SR-B1; GenBank accession no. NM_031541; 141 bp) forward, 5′-TACTTGTCCGTCTACT-3′ and reverse 5′-CGTGTCATTGTCATTG-3′; 18S (GenBank accession no. M11188; 205 bp) forward, 5′-TTCAGCCACCCGAGAT-3′ and reverse 5′-GCTTATGACCCGCACTTA-3′. The products were analyzed using the CT value and all values were normalized against the 18S mRNA level. The final result was calculated using the 2^−ΔΔCt^ method.

### Statistical analysis

All the data are presented as the mean ± standard deviation (n=6). Statistical analysis was performed using the SPSS version 17.0 statistical software (SPSS, Inc., Chicago, IL, USA). A one-way analysis of variance was used to analyze differences between the biochemical parameters among the groups, followed by Dunnett’s significant post-hoc test for pairwise multiple comparisons. P<0.05 was considered to indicate a statistically significant difference.

## Results

### Effects of C, P and CP administration on the growth parameters of rats fed on a HFD

As shown in Table I, no significant difference between the initial body weight, final body weight, body weight gain, food intake and relative liver weight among the groups was identified.

### Effects of C, P and CP administration on the serum lipid levels of rats fed on a HFD

Table II shows the serum levels of TC, TG, HDL-C and LDL-C from rats in each group. TC, TG, HDL-C and LDL-C levels in the H group were significantly different compared with the N group. The levels of TC, TG and LDL-C in the H + C and H + CP groups were markedly lower compared with those in the H group, whilst the level of HDL-C in the H + CP group was markedly elevated compared with the H group. In addition, compared with the N group, the levels of ApoAI and ApoB were significantly different in the H group. Administration of CP significantly increased the levels of ApoAI.

### Effects of C, P and CP administration on the hepatic and fecal lipid levels of rats fed on a HFD

The hepatic TC and TG levels were significantly increased in the H group compared with the N group, whilst the hepatic TC and TG levels in the H + C group and H + CP group were significantly decreased compared with the H group (Table III). The fecal TC, TG and TBA levels were also significantly increased in the H group compared with the N group. In addition, compared with the H group, the TC, TG and TBA levels in the H + C and H + CP groups were significantly increased. Furthermore, the TC, TG and TBA levels in the H + CP were significantly different from those in the H + C group.

### Effects of C, P and CP administration on the activity of serum LCAT and hepatic CYP7A1 in rats fed on a HFD

As shown in Table IV, compared with the N group, serum LCAT activity was markedly decreased in the H group. Administration of CP markedly increased the LCAT activity compared with the H or H + C group. Compared with the N group, hepatic CYP7A1 activity was significantly increased in the H group. Administration of CP clearly increased the CYP7A1 activity compared with the H or H + C group.

### Effects of C and CP administration on the hepatic mRNA levels of rats fed on a HFD

**Figure 1:**
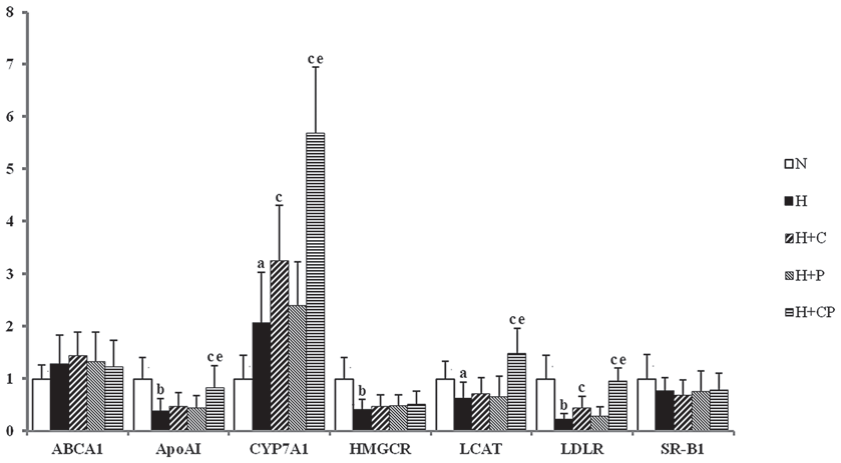
Analysis of hepatic mRNA levels using quantitative polymerase chain reaction of rats in each group. The relative mRNA levels are presented as the mean ± standard deviation obtained from six rats in each group. ^a^P<0.05, ^b^P<0.01, vs. N group; ^c^P<0.05, vs. H group; ^e^P<0.05, vs. H + C group. N, normal control; H, high-fat diet control; H + C, high-fat diet plus curcumin; H + P, high-fat diet plus piperine; H+CP, high-fat diet plus curcumin plus piperine; ABCA1, ATP-binding cassette transporter A1; ApoA1, apolipoprotein AI; CYP7A1, cholesterol 7α-hydroxylase; HMGCR, HMG-CoA reductase; LCAT, lecithin cholesterol acyltransferase; LDLR, low-density lipoprotein receptor; SR-B1, scavenger receptor B1.

qPCR analysis was performed to measure the mRNA levels in the rat livers in each group (Fig. 1). Compared with the H and H + C groups, administration of CP caused a marked increase in ApoAI, CYP7A1, LCAT and LDLR mRNA levels. However, no significant differences were observed between the groups for other mRNAs associated with cholesterol metabolism.

## Discussion

In the present study, a significant increase in TC, TG and LDL-C levels, as well as a decrease in HDL-C levels in the H group was observed, indicating that HLP in SD rats was successfully induced via administration of a HFD. Administration of curcumin plus piperine resulted in a significant decrease in the serum TC, TG and LDL-C levels compared with the H and H + C groups. Notably, the results from the present study demonstrated that curcumin did not significantly alter the HDL levels, which is consistent with the results from Kim *et al* (16); however, they are inconsistent with the results from Arafa *et al* (12). In addition, only curcumin plus piperine significantly increased the levels of ApoAI and HDL-C, as well as LCAT activity.

Notably, only curcumin plus piperine significantly increased the levels of HDL-C. Previous studies have demonstrated that HDL is synthesized through a complex pathway (31). HDL assembly initially involves the cell surface ABCA1 transporter-mediated transfer of phospholipids and cholesterol to the extracellular lipid-poor ApoAI. The plasma compartment of HDL particles is then remodeled by the esterification of cholesterol via the LCAT enzyme, the exchange between HDL and other lipoproteins and the putative transfer of cellular cholesterol to the growing particles by SR-B1, which is also an important receptor in cholesterol metabolism (32). In the present study, no significant differences in ABCA1 and SR-B1 mRNA levels were observed. Previous studies have demonstrated that the increase in LCAT activity and ApoAI levels may contribute to an increase in HDL-C concentrations (33–35). However, in the present study, a significant increase in the level of ApoAI and the activity of LCAT was observed in the H + CP group. Therefore, to further explain the increase of serum HDL-C level in rats in the H + CP group, the expression of ApoAI and LCAT mRNA was analyzed. It was found that the mRNA levels of LCAT and ApoAI significantly increased following administration of curcumin and piperine, suggesting a co-ordinated regulatory mechanism of ApoAI and LCAT mRNA expression. This suggests that the higher level of HDL-C in rats in the H + CP group may be due to alterations in the mRNA expression of ApoAI and LCAT. Therefore, this indicates that curcumin plus piperine increases cholesterol efflux to HDL particles by elevating the ApoAI and LCAT mRNA levels, resulting in a significant increase in serum HDL-C levels.

Cholesterol metabolism is primarily conducted in the liver. The conversion of cholesterol to bile acids in the liver is an important pathway for the elimination of cholesterol from the body (36), which accounts for ~50% of daily cholesterol excretion (37). CYP7A1 is a liver-specific enzyme that catalyzes the rate-limiting step in the biosynthesis of bile acid from cholesterol (38). An increase in hepatic CYP7A1 gene expression may contribute to the enhancement of CYP7A1 activity and therefore increase the fecal bile acid levels. This may lead to an increase in the quantity of cholesterol that is excreted out of the body and thus lead to a decrease in serum cholesterol levels. In the present study, the expression level of CYP7A1 was found to be markedly upregulated by high plasma cholesterol in the H group, suggesting that the synthesis of bile acid is positively associated with plasma cholesterol levels (39). Increased CYP7A1 mRNA levels were also observed in rats treated with curcumin, which is in accordance with the results obtained by Kim *et al* (16). Furthermore, the present study found that curcumin plus piperine increased CYP7A1 activity and gene expression to a greater extent than curcumin alone. This demonstrated that curcumin plus piperine increased the rate of bile acid production, which is the precursor molecule for cholesterol synthesis and, therefore, curcumin and piperine induce a more efficient removal of excessive cholesterol from the blood. However, a certain amount of cholesterol may also be transformed into fecal cholesterol by microbes in the intestine and excreted through the feces (26). The results from the present study demonstrated that fecal TC and TG levels were greater in rats administrated with curcumin plus piperine compared with rats in the other groups. These results are consistent with the previous studies that found a curcumin-mediated increase in fecal excretion of cholesterol (16,40).

The mRNA levels of genes associated with cholesterol synthesis were measured in the present study. HMGCR is a rate-limiting enzyme in cholesterol synthesis (14,41). The present study demonstrated that the mRNA expression of HMGCR in the H group was inhibited due to feedback inhibition induced by an increase in exogenous cholesterol. In addition, no significant differences were observed among the three groups, including the H, H + C and H + CP groups, suggesting neither C nor CP affected the synthesis of cholesterol. LDLR is an important receptor that mediates the clearance of LDL-C in the blood. It has previously been demonstrated that curcumin treatment upregulated LDLR mRNA levels in human hepatoma HepG2 cells (42,43) and SD rats (16). The present study revealed that curcumin plus piperine had a more significant effect on LDLR mRNA expression levels than curcumin alone, leading to a marked decrease in LDL-C levels.

In combination, the results from the present study demonstrated that piperine was able to enhance the hypocholesterolemic effect of curcumin via modulating the expression of a number of genes involved in cholesterol metabolism. In the presence of piperine, curcumin was found to increase the mRNA expression levels of ApoAI, CYP7A1, LCAT and LDLR, leading to an increase in the transformation of cholesterol to bile acid, an increase in HDL-C levels and a reduction of LDL-C levels. Therefore, this suggests that redundant TC in the blood may be removed by HDL and transferred to the feces. It has previously been demonstrated that the combination of piperine and curcumin was able to increase bioavailability of curcumin (17,20). The present study demonstrated that administration of piperine alone (5 mg/kg/day) had no significant hypocholesterolemic effect; however, administration of piperine plus curcumin markedly increased the hypocholesterolemic effect, via upregulation of the activity and gene expression levels of ApoAI, CYP7A1, LCAT and LDLR. This suggests that the enhanced ability of curcumin plus piperine compared with curcumin alone in lowering serum cholesterol level is due to an increase in the bioavailability of curcumin.

In conclusion, co-administration of curcumin plus piperine was found to decrease the serum and liver levels of TC, TG and LDL-C, as well as increase the fecal levels of TC, TG and TBA to a greater extent compared with curcumin alone. In addition, curcumin plus piperine markedly increased the levels of HDL-C. Furthermore, compared with administration of curcumin alone, administration of curcumin plus piperine resulted in a more significant upregulation of the activities and gene expression levels of ApoAI, LCAT, CYP7A1 and LDLR. Therefore, the results from the present study demonstrate that co-administration of curcumin plus piperine is better than administration of curcumin alone against HLP, providing a promising combination for the treatment of HLP.

## Figures and Tables

**Table I:** Growth parameters of rats fed on a HFD.

Parameter	N	H	H + C	H + P	H + CP
Initial body weight (g)*	314.33±6.38	307.15±5.25	305.18±5.84	303.98±8.12	306.65±8.80
Final body weight (g)**	369.58±20.85	358.83±19.69	360.05±13.68	365.93±11.04	354.65±9.79
Body weight gain (g)	55.25±14.42	51.68±15.23	54.87±12.10	54.37±12.18	48.00±9.34
Food intake for 4 weeks (g)	393.77±12.22	386.87±11.34	374.25±13.77	382.44±12.16	361.32±12.09
Relative liver weight (g/100 g of body weight)	3.73±0.26	3.65±0.38	3.36±0.10	3.44±0.18	3.35±0.09

The results are presented as the mean ± standard deviation obtained from six rats in each group.

* Body weight at the beginning of the 4-week oral administration of C, P or CP.

** Body weight at the end of the 4-week oral administration of C, P or CP.

HFD, high-fat diet; N, normal control; H, HFD control; H + C, HFD plus curcumin; H + P, HFD plus piperine; H + CP, HFD plus curcumin plus piperine.

**Table II:** Effects of C, P and CP on the serum lipid levels of rats fed on a HFD.

Parameter	N	H	H + C	H + P	H + CP
TC (mmol/l)	4.02±0.96	7.16±1.59^b^	5.18±1.47^c^	6.63±1.89	4.52±1.36^d^,^e^
TG (mmol/l)	1.94±1.05	4.28±1.17^b^	3.07±1.30^c^	4.07±1.16	1.96±1.33^d^,^e^
HDL-C (mmol/l)	2.85±0.58	1.70±0.16^a^	1.84±0.51	1.61±0.24	2.88±0.46^d^,^e^
LDL-C (mmol/l)	1.16±0.66	5.34±0.52^b^	3.30±1.18^c^	5.01±0.43	1.64±0.77^d^,^e^
ApoAI (g/l)	0.063±0.006	0.018±0.006^b^	0.024±0.010	0.021±0.009	0.064±0.011^d^,^e^
ApoB (g/l)	0.075±0.017	0.112±0.010^a^	0.109±0.011	0.114±0.019	0.107±0.012

The results are presented as the mean ± standard deviation obtained from six rats in each group.

a P<0.05,

b P<0.01 vs. N group;

c P<0.05,

d P<0.01 vs. H group;

e P<0.05, vs. H + C group.

HFD, high-fat diet; TC, total cholesterol; TG, triglyceride; HDL-C, high-density lipoprotein cholesterol; LDL-C, low-density lipoprotein cholesterol; Apo, apolipoprotein; N, normal control; H, HFD control; H + C, HFD plus curcumin; H + P, HFD plus piperine; H + CP, HFD plus curcumin plus piperine.

**Table III:** Effect of C, P and CP administration on hepatic and fecal lipid levels of rats fed on a HFD.

Parameter	N	H	H + C	H + P	H + CP
TC (μmol/g liver)*	9.97±3.12	34.76±4.31^b^	20.38±2.86^c^	29.91±3.17	12.52±2.27^d^^e^
TG (μmol/g liver)*	17.26±3.78	40.35±4.84^b^	27.90±3.06^c^	36.46±4.38	22.56±3.44^d^^e^
TC (μmol/g of feces/day)#	5.69±1.47	11.10±2.29^b^	14.77±2.51^c^	12.05±2.40	17.33±3.16^d^^e^
TG (μmol/g of feces/day)#	1.71±0.36	3.84±2.25^b^	6.16±2.37^c^	4.92±2.12	7.05±2.40^d^^e^
TBA (μmol/g of feces/day)#	169.21±30.8	218.10±16.65^a^	241.82±20.50^c^	225.63±18.47	266.44±29.43^d^^e^

The results are presented as the mean ± standard deviation from six rats in each group.

* Hepatic lipids and

# Fecal lipids.

a P<0.05,

b P<0.01, vs. N group;

c P<0.05,

d P<0.01 vs. H group;

e P<0.05,

f P<0.01 vs. H + C group.

HFD, high-fat diet; TC, total cholesterol; TG, triglyceride; TBA, total bile acid; N, normal control; H, HFD control; H + C, HFD plus curcumin; H + P, HFD plus piperine; H + CP, HFD plus curcumin plus piperine.

**Table IV:** Effect of C, P and CP administration on the activity of CYP7A1 and LCAT in rats fed on a HFD.

Parameter	N	H	H + C	H + P	H + CP
LCAT (nmol/h/ml)	16.3±2.01	9.68±1.37^b^	10.29±1.17	9.70±0.87	13.95±1.00^d^^e^
CYP7A1 (nmol/h/mg protein)	1.12±0.39	1.77±0.46^a^	2.23±0.75^c^	1.75±0.48	3.04±0.61^d^^e^

The results are presented as the mean ± standard deviation obtained from six rats in each group.

a P<0.05,

b P<0.01, vs. N group;

c P<0.05,

d P<0.01, vs. H group;

e P<0.05,

f P<0.01, vs. H+C group.

LCAT, lecithin cholesterol acyltransferase; CYP7A1, cholesterol 7α-hydroxylase; HFD, high-fat diet; N, normal control; H, HFD control; H + C, HFD plus curcumin; H + P, HFD plus piperine; H + CP, HFD plus curcumin plus piperine.
